# Trends in and contributions to entrepreneurship research: a broad review of literature from 1996 to June 2012

**DOI:** 10.1007/s11192-013-1203-5

**Published:** 2013-12-15

**Authors:** Tainyi Luor, Hsi-Peng Lu, Hueiju Yu, Kuoliang Chang

**Affiliations:** 1 National Taiwan University of Science and Technology, New Taipei, Taiwan, ROC; 2 Chinese Culture University, Taipei, Taiwan, ROC; 3 International Bills Finance Corporation, Taipei, Taiwan, ROC

**Keywords:** Entrepreneur, Contribution research, Literature review, Trend, Citation analysis

## Abstract

This article, which began as an effort to gauge trends in and contributions to the broad field of “entrepreneur/entrepreneurship,” reviews 5,476 academic articles on entrepreneurship that were published in 522 Social Sciences Citation Index and Science Citation Index journals from 1996 to June 2012. This survey identifies keywords and conducts a review to search for and identify related articles in the Institute for Scientific Information Web of Science database. We then present our findings, including the number of publications by year, categorization of article types, main academic journals, authors, and most-cited articles. The citation counts for authors, journals, and articles are also analyzed. This study indicates that the number of articles related to the keyword entrepreneur increased from 1996 to the end of 2011, which is a sign of an upward trend in the influence of entrepreneurs. Entrepreneur research fascinated numerous scholars during the study period covering 16.5 years. In particular, researchers from the USA, England, Canada, Germany, and the Netherlands have made the most contributions to this field. This literature review provides evidence that the concept of entrepreneur attracted academic researchers, resulting in significant contributions to the field of entrepreneur research.

## Introduction

Entrepreneurship has been known to serve an important function in job creation, economic growth, and development of various geographic entities, from villages to regions and even to entire countries. Scholars have defined entrepreneurship in numerous ways (Landstrom et al. 2012). For instance, Shane and Venkataraman (2000) provided a comprehensive definition: “The field of entrepreneurship is the scholarly examination of how, by whom, and with what effects opportunities to create future goods and services are discovered, evaluated and exploited.” These authors claimed that entrepreneurship involves sources of as well as the processes of discovery, evaluation, and exploitation of opportunities. Davidsson (2005) added that entrepreneurship could have either been related to the “entrepreneurial individual” or framed as the creation and running of one’s own firm. Hitt et al. (2011) also defined entrepreneurship as a set of individuals who discover, evaluate, and exploit these opportunities. In the academic literature, numerous studies have introduced the attributes and domain of entrepreneurship or tried to explore its streams. Shane and Venkataraman (2000) argued that previous entrepreneurship research was only focused on small businesses or new firms, disregarding the research field as a unique conceptual domain. They also stated that people have had trouble identifying the unique contributions of entrepreneurship research to the broader domain of business studies, which undermines the legitimacy of entrepreneurship as a research field. Therefore, the authors proposed an integrated framework for entrepreneurship and attempted to help entrepreneurship researchers recognize the relationship among the multitude of necessary but insufficient factors that comprise this field. Their ultimate goal was to improve the quality of empirical and theoretical research on entrepreneurship.

Busenitz et al. (2003) used the keywords “entrepreneur” (entrepreneurial and entrepreneurship), “small business” (emerging business), “new venture” (emerging venture), and “founder(s)” to search for entrepreneurship-related articles in the ABI/Inform database. They applied boundary and exchange concepts to examine 97 entrepreneurship articles published from 1985 to 1999 in seven leading management journals, namely, Academy of Management Journal, Academy of Management Review, Strategic Management Journal, Journal of Management, Organization Science, Management Science, and Administrative Science Quarterly. The articles were used as a basis to evaluate the emergent academic field of entrepreneurship and to understand its progress and potential better. The highly permeable boundaries of entrepreneurship enable intellectual exchange with other management areas but discourage the development of an entrepreneurship theory and deter the legitimacy of the field. The authors argued that focusing on entrepreneurship research at the intersection of the constructs of individuals, opportunities, modes of organization, and the environment can define the field and enhance its legitimacy. Their regression analysis results demonstrate a positive trend for entrepreneurship publication in management journals, although the percentage of entrepreneurship articles in their findings remains low. Furthermore, a number of comprehensive reviews of entrepreneurship literature (Schildt et al. 2006; Cornelius et al. 2006; Denis et al. 2006; Brian et al. 2011; Landstrom et al. 2012; Meyer et al. 2013) gauge the intellectual origins and structure of the entrepreneurship domain.

Based on a systematic review of 57 high-quality studies containing 87 separate but relevant analyses, Mirjam and Versloot (2007) stated that entrepreneurs have a very important function in the economy. Bhupatiraju et al. (2012) also applied network analysis to a citation database that combines key references in the fields of entrepreneurship, innovation studies, and science and technology studies. They found that citations among the three fields are relatively scarce compared with those within each field. A cluster analysis of the publications in the database generates a partition that is largely the same as the a priori division into the three fields. The authors considered this result as evidence that the three fields have developed mainly on their own and in relative isolation from one another. Using a “main path” analysis aimed at outlining the main research trajectories in the fields, the researchers argued that entrepreneurship developed relatively late and has shown a trajectory that remains in its infancy.

Another recent academic study involves Kano’s theory of attractive quality (Löfgren and Witell 2008). The authors systematically reviewed the subsequent development of Kano’s theory and selected papers from the ABI/Inform, Academic Search Elite, Business Source Elite, Emerald, and Institute for Scientific Information (ISI) databases by employing a snowball technique and using reference lists to collect related studies. They synthesized their findings and offered suggestions for research themes of quality dimensions after reviewing 33 papers related to Kano’s theory. Furthermore, their study also revealed several interesting developments with respect to methodological issues that promote the development of the theory of attractive quality.

The academe has recently identified the rising significance of indices, such as the Sciences Citation Index (SCI) or Social Sciences Citation Index (SSCI), which supposedly assess the “impact factors” of researchers and individual publications on their respective individual fields. SSCI or SCI includes the collective bibliography sections of selected prestigious academic journals. The impact factor is measured each time a reference found in the bibliography of an SCI- or SSCI-weighted journal is cited by another author. Bhupatiraju et al. (2012) also focused on citations, arguing that citations are indications of intellectual influence and can thus be used as “paper trails” of the flow of ideas between and within the three fields. Although citation analysis is well accepted by numerous researchers as a valid analytical tool, data limitations must be met with caution. Citations may depend on both the actual flow of ideas and specific habits and norms, which potentially differ among fields. For example, extremely long reference lists may be customary in several fields, but other fields have shorter lists. Citation patterns may be influenced by “strategic” motivations. For example, a specific paper or author may be cited (or not cited) to increase the probability of publication, or citations may be affected by personal friendships or dislikes. Regardless of all these influences, researchers still depend on citation data for their analysis. Therefore, even if a citation analysis cannot shed full light on their topic, researchers argue that such analysis should provide a major addition to what they know. Our study began as an effort to identify the main researchers in the field of entrepreneurship and calculate their “contributions” along with the number of times that they had been published and cited by others, as stated in the ISI Web of Science (WOS) database. The credits for each publication from individual researchers are calculated. This calculation, which is based on the SCI/SSCI, is used to compute for the credit of individual journals and individual researchers on entrepreneurship literature.

Other studies referenced or discussed entrepreneurship research in terms of its development and provided background on the central issue of legitimacy. In his survey of tenured entrepreneurship scholars in leading universities, MacMillan (1991, 1993) found that publications indicative of the highest scholarly competence include the Administrative Science Quarterly, Academy of Management Review, Academy of Management Journal, Journal of Business Venturing, and Strategic Management Journal. Harrison and Leitch (1996) found that entrepreneurship studies published in management journals from 1987 to 1993 represent a small percentage of all published entrepreneurship studies and that the majority of such research is published in journals dedicated to entrepreneurship and small businesses. The authors also advised that entrepreneurship scholars may become more self-referential and inward directed. This change is a consequence of the field’s reliance on dedicated entrepreneurship journals at the expense of intellectual development achieved through the external legitimization of its tenets in publications for various management areas.

**Fig. 1:**
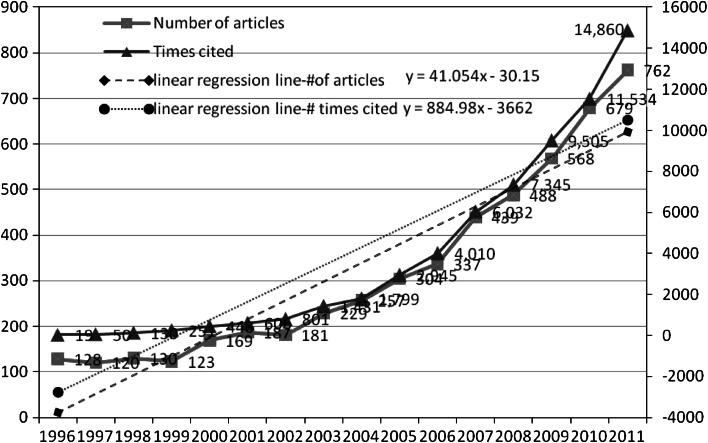
Number of articles and times cited publications (1996–2011)

Despite the existence of several previous studies on entrepreneurship research, limited information is available on the stream of this research field. Unlike the aforementioned studies that are related to entrepreneurship, the present study attempts to explore the trends in and contributions to the field of entrepreneurship as well as to shed light on the field of entrepreneurship research from different perspectives. We conduct a wide-ranging literature review and survey 5,476 entrepreneur articles that were published in 522 journals from 1996 to June 2012, considering that entrepreneurship research covers a broader domain of business studies. In conducting a keyword search of the WOS databases to find 5,476 entrepreneurship-related publications in 522 academic journals from 1996 to June 2012 (see Fig. 1), we adopt the methods proposed by the following researchers: MacArthur et al. (2001), who defined the use of technology in teaching disabled students; Zou (2005), who reviewed literature relating to advertisement; Latchem (2006), who adopted content analysis to quantify the foci and occurrence of topics published in the British Journal of Educational Technology between 2000 and November 2005; and Luor et al. (2008), who reviewed the literature relating to computer-assisted learning. The authors of the present study first describe their data-gathering methods and then present their findings.

## Research methods

### Scope of study

The first internet boom began in April 1996 when Yahoo was listed on the US stock market. During this time, over nine million internet hosts emerged, which grew to more than 16 million by 1997. Some entrepreneurs started to set up their business base on the Internet (Boomtime 2013) and that may trigger more studies with entrepreneurship. Therefore, our study selected 1996 as the start year for our analysis. We conducted a keyword search using two entrepreneurship-related terms or keywords in the WOS databases to identify 5,476 entrepreneur-related articles in 522 academic journals from 1996 to June 2012. We first used the keyword “entrepreneur” (articles related to entrepreneurship will also be included) in searching for the article topic. The searched categories are arbitrarily limited to six types: (1) management, (2) business, (3) social psychology, (4) economics, (5) planning development, and (6) business finance. Articles were then assigned to one of the 37 subject areas (Table 1). Among the 37 subject areas categorized by the ISI WOS, “Business Economics,” “Public Administration,” “Engineering,” “Operations Research Management Science,” “Social Sciences Other Topics,” and “Psychology” are the six subject areas that were found to be the most related to entrepreneurship.

**Table 1:** Top six subject areas and numbers of articles (1996–Jun. 2012)

Subject area	No. of articles	% of 5,476 articles (%)
Business economics		
Management		21.9
Business		21.8
Economics		15.5
Planning development		5.7
Business finance		2.4
Psychology social		0.4
	5,219	67.7
1. Public administration	694	9.0
2. Engineering	272	3.5
3. Operations research management science	248	3.2
4. Social sciences other topics	248	3.2
5. Psychology	190	2.5
6. Other areas		
Geography		2.2
Environmental sciences		2.1
Government law		1.7
Urban studies		1.1
Mathematical methods in social sciences		0.6
Others		3.2
	835	10.8

Total subject areas: 37. The number of articles displayed may be greater than the listed record Count, because that some articles may across multiple subject areas

### Calculation of authorial “contribution”

The authors used the “times cited” counts acquired from the ISI WOS database to reveal the “contributions” of authors and individual publications to the field. The resulting measurement scale calculated the number of times an article was published and subsequently cited within the ISI WOS database.

The number of times a journal article was cited is indicative of the contribution of that article because the author(s) of the article evaluated the cited works based on their value to his/her own study and to the progress of the research field in general (Chandy and Williams 1994; Cote et al. 1991). Consequently, the number of times that a published journal article was cited in the literature is an appropriate scale to evaluate the influence of that article on the literature. Alternatively, citation analysis occasionally results in differences of opinion, such as bias that favors extensive auto-citations or methodological articles of authors. In the academe, citation analysis is commonly recognized as a credible tool for determining the overall influence of an article on the field. In this literature review, the number of times every author was cited (impact) on the 5,476 entrepreneur research articles was acquired from the ISI WOS database.

The number of times an article is cited indicates that the author(s) of the article measured the cited works notable to their own study and to the advancement of their research field (Chandy and Williams 1994; Cote et al. 1991). Thus, the number of times a published journal article is cited is an appropriate measure of the influence of the article on the literature.

In the academe, the SCI/SSCI is commonly acknowledged as an acceptable weight of article impact. The impact of each author on the 5,476 entrepreneur research articles were calculated in this study. The sum of the SCI/SSCI citation count of an author is equivalent to his or her collective impact on the entrepreneurship research literature. For example, the total SCI/SSCI citation count of an author is the sum of that of two articles. If an author has published two articles in the 5,476-article pool, then the SCI/SSCI citation count of an article reveals its impact. The total SCI/SSCI citation count of an author determines his or her impact on the entrepreneurship research literature. The SCI/SSCI citations for each article in the aforementioned pool were obtained from the ISI WOS database. In this article, the Web-based SCI/SSCI database of the ISI was used to calculate the impact of each article and its author(s). For the calculation of credited contributions to a publication, a single author for an article was credited with one (1) count. If an article has two authors, then each author was credited with 0.5. The same rule for articles with more authors was applied to calculate the credited contribution for each author. The total credited contributions of an author were summed up for all articles that he or she has authored or co-authored within the 5,476 entrepreneurship article pool.

#### Application of Lotka’s law and Bradford’s law

Lotka’s law of scientific productivity (1926) and Bradford’s law of bibliographic scattering (1934) are two familiar empirical laws in information science.

In 1926, Lotka introduced an empirical law to calculate the scientific productivity of authors in the field of chemistry. In Lotka’s law, if *x* authors publish exactly one paper each, then the number of authors a_b_ contributing b papers would be given by

$$ a_{b} = x/b^{ 2} \quad {\text{for}}\,b = 1,{ 2}, \, \ldots $$

In this paper, we adopted the Chi square test (Radhakrishnan and Kernizan 1979) to verify whether entrepreneurship papers published in the SCI/SSCI from 1996 to June 2012 satisfied Lotka’s law. First, considering that the most number of publications by one author is 56, we must calculate the number of authors for five groups: *a*_1_ (number of authors with one publication), *a*_2_ (number of authors with two publications), *a*_3_ (number of authors with three publications), *a*_4_ (number of authors with four publications) …, and *a*_56_ (number of authors with 56 publications). Second, the Chi square test for varying *x*/1^2^, *x*/2^2^, *x*/3^2^ …, and *x*/56^2^ for groups *a*_1_, *a*_2_, *a*_3_, *a*_4…_ and *a*_56_ were summed up and tested.

In Bradford’s Law, if a complete literature search is conducted on several subjects covering a particular period, then the literature is scattered in a normal pattern over a large number of sources. When these sources are ranked in descending order of productivity, with the journals that published the most articles at the top of the list and those that published the fewest articles at the bottom, sources can be partitioned into main periodicals particularly devoted to the subject and into several groups or zones containing the same number of articles as the core, where the number of periodicals in the core and succeeding zones is marked as

$$ 1:m:m^{ 2} : \, \ldots $$

for some constant *m* (Chen and Leimkuhler 1986).

Journals were divided into three groups to verify the applicability of Bradford’s law: a relatively small group of core journals that published approximately one-third of all articles (1st group), a large group of journals but containing the same number of articles as the first group (2nd group), and an even larger group of journals but published the equivalent number of articles (3rd group) as the other groups.

## Research results

### Frequency of entrepreneurship research in journals

Figure 1 indicates the citation count and the overall number of entrepreneurship articles published in academic journals from 1996 to the end of 2011. The overall number of articles increased annually, and we derive a liner regression equation as *Y* = 41.054*X* − 30.15, which indicates that that trend is positive annually. The overall citation count also increased annually, and we derive a liner regression equation as *Y* = 884.98*X* – 3,662. Both slope values were calculated by assuming a linear function.

**Fig. 2:**
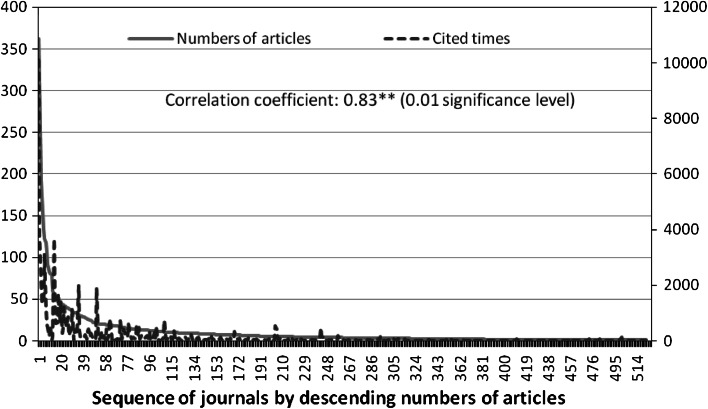
Number of articles and times cited by journals (1996–Jun. 2012)

A total of 522 journals were found to be related to entrepreneurship research, and these journals published entrepreneurship research articles between 1996 and June 2012. The number of articles and credited citation count for these journals are presented in Table 2. Approximately 43.4 % of the 5,476 entrepreneurship-related articles published in the 522 journals from 1996 to June 2012 appeared in the top 20 journals (Table 2). The top three journals, namely, Journal of Business Venturing (362 articles or 6.6 %), Small Business Economics (309 articles or 5.6 %), and Entrepreneurship Theory and Practice (192 articles or 3.5 %), published over 190 articles each. Moreover, 502 other journals published less than 46 entrepreneurship-related articles. The number of articles published by the journals in descending order is shown in Fig. 2 (in solid line).

**Table 2:** Most frequent venues of journals for entrepreneur publications among 522 journals (1996–Jun. 2012)

	Journal title	No. of articles (% of total)	Credit of cited times (% of total)
1.	Journal of business venturing	362 (6.6)	10,096 (14.8)
2.	Small business economics	309 (5.6)	3,772 (5.5)
3.	Entrepreneurship theory and practice	192 (3.5)	2,065 (3.0)
4.	Entrepreneurship and regional development	172 (3.1)	1,422 (2.1)
5.	Journal of small business management	133 (2.4)	1,314 (1.9)
6.	Research policy	122 (2.2)	3,107 (1.6)
7.	Technovation	117 (2.1)	1,156 (1.7)
8.	International small business journal	106 (1.9)	596 (0.9)
9.	Journal of business ethics	91 (1.7)	464 (0.7)
10.	African journal of business management	82 (1.5)	151 (0.3)
11.	Strategic entrepreneurship journal	80 (1.7)	567 (0.8)
12.	Forbes	79 (1.5)	3 (0.0)
13.	International journal of technology management	60 (1.1)	140 (0.2)
14.	Strategic management journal	56 (1.0)	3,654 (5.4)
15.	Organization studies	52 (0.9)	994 (1.5)
16.	Journal of management studies	50 (0.9)	607 (0.9)
17.	Harvard business review	49 (0.9)	638 (0.9)
18.	Organization science	47 (0.9)	1,699 (2.5)
19.	Journal of business research	46 (0.8)	657 (1.0)
20.	Management science	46 (0.8)	1,427 (2.1)
	502 Journals	<46 (56.6 %)	33,754 (49.4 %)

The credited citation count for each journal is also calculated and listed on the right column of Table 2. The top 20 journals that published the most number of articles also contributed 34,527 citation counts (50.6 %) in the 5,476 entrepreneurship article pool. The other 502 journals contributed 33,754 credit citation counts (49.4 %). Furthermore, journals such as the Strategic Management Journal, Organization Science, and Management Science published a lower percentage of articles but contributed a higher percentage of credited citation count. The credited citation count by journal is shown in Fig. 2 (in dotted line). The correlation coefficient between the number of articles and credited citation count of journals was 0.83 and was tested to be significant based on linear assumption. Nevertheless, several journals published fewer articles but contributed a significant credited citation count. These journals include the following: Academy of Management Review [Number of articles: 21 (0.4 % of article pool), Credited citation count: 1,898 (2.8 % of article pool)], Academy of Management Journal [Number of articles: 31 (0.6 % of article pool), Credited citation count: 2,009 (2.9 % of article pool)], Journal of International Business Studies [Number of articles: 42 (0.8 % of article pool), Credited citation count: 1,207 (1.8 % of article pool)], Administrative Science Quarterly [Number of articles: 10 (0.2 % of article pool), Credited citation count: 720 (1.1 % of article pool)], Journal of Finance [Number of articles: 19 (0.3 % of article pool), Credited citation count: 795 (1.2 % of article pool)], and Journal of Financial Economics [Number of articles: 17 (0.3 % of article pool), Credited citation count: 734 (1.1 % of article pool)].

### Chi square tests of Bradford’s law for the number of entrepreneurship research articles in journals

The plot of the number of articles versus the sequences of journals by number of entrepreneurship articles is shown in solid line in Fig. 2. As anticipated, several primary journals published a larger number of entrepreneurship articles. Table 3 provides a result of the Chi square test of Bradford’s Law for the entrepreneurship literature. The literature was divided into three groups: 1st, 2nd, and 3rd. The 1st group has 12 journals that published 1,845 articles, the 2nd group has 63 journals that published 1,809 articles, and the 3rd group has 447 journals that published 1,862 articles. The Chi square values clearly show that Bradford’s original suggestion of 1:*m*:*m* ^2^ does not appropriately apply. However, after Chi square testing, the prediction of 1:*m*:*m* ^2.2^ is quite close.

**Table 3:** Chi squared test of Bradford’s Law

Group	No. of articles	No. of journals
1st	1,845	12
2nd	1,809	63
3rd	1,822	447

Chi squared value for 1:*j*:*j* ^2^: 40.86. Chi squared value for 1:*j*:*j* ^2.2^: 0.41

### Frequency of entrepreneurship research by country and institution

Table 4 presents the top 14 countries that published 79.5 % of the 5,476 entrepreneurship-related articles: the USA, England, Canada, Germany, Netherlands, Spain, Sweden, Australia, Italy, China, France, Scotland, Belgium, and Switzerland. As shown in the upper part of Table 5, 13 institutions published 869 articles or 15.9 % of the 5,476 articles: Indiana University, Harvard University, Erasmus University, University of Nottingham, Babson College, University of Minnesota, Max Planck Institute of Economics, Rensselaer Polytechnic Institute, University of Illinois, University of North Carolina, University of Amsterdam, University of Colorado, and Stanford University.

**Table 4:** Most frequent venues of top 100 countries for entrepreneur publications among 522 journals (1996–Jun. 2012)

	Top 100 countries	No. of articles	% of total
1.	USA	2,293	32.9
2.	England	763	11.0
3.	Canada	403	5.8
4.	Germany	352	5.1
5.	Netherlands	319	4.6
6.	Spain	207	3.0
7.	Sweden	201	2.9
8.	Australia	183	2.6
9.	Italy	171	2.5
10.	Peoples R China	161	2.3
11.	France	145	2.1
12.	Scotland	133	1.9
13.	Belgium	105	1.5
	Switzerland	105	1.5
	86 Countries	<105	20.5

**Table 5:** Most Active 100 Institutional Contributors and Researchers (1996–Jun. 2012)

	Institutional contributors	Number of articles	% of 5,476 articles
1.	Indiana univ	108	2.0
2.	Harvard univ	83	1.5
3.	Erasmus univ	81	1.5
4.	Univ Nottingham	76	1.4
5.	Babson coll	68	1.2
6.	Univ Minnesota	66	1.2
7.	Max planck inst econ	64	1.2
8.	Rensselaer polytech inst	58	1.1
9.	Univ illinois	55	1.0
10.	Univ n Carolina	54	1.0
11.	Univ Amsterdam	53	1.0
12.	Univ Colorado	52	0.9
13	Stanford univ	51	0.9
	87 Institutions	<51	84.1

	Researcher	Number of articles	% of 5,476 articles
1.	Wright, M	56	1.02
2.	Shepherd, DA	44	0.8
3.	Audretsch, David	39	0.71
4.	Shane, S	36	0.66
5.	Zahra, SA	33	0.60
6.	Baron, RA	26	0.47
7.	Acs, ZJ	24	0.44
8.	Parker, SC	24	0.44
9.	Westhead, P	22	0.40
10.	Thurik, R	20	0.37
	7,395 authors	<20	94.08 %
	Total Authors: 7,405		

### Main individual contributors and most frequently published credited authors of entrepreneurship

**Fig. 3:**
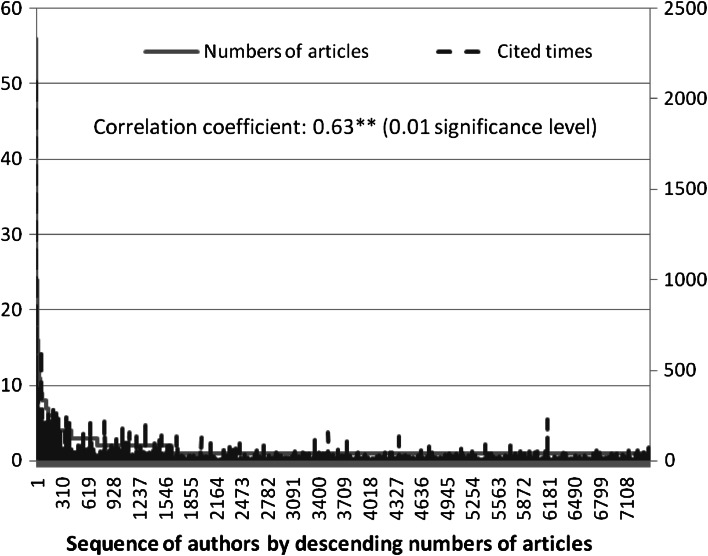
Number of articles and times cited by authors (1996–Jun. 2012)

As shown in the lower part of Table 5, 10 scholars among the authors of the 5,476 articles have published over 20 publications, and 7,395 published less than 20 publications from 1996 to June 2012. The 10 authors who published the most number of entrepreneurship-related articles are M. Wright, D.A. Shepherd, David Audretsch, S. Shane, S.A. Zahra, R.A. Baron, Z.J. Acs, S.C. Parker, P. Westhead, and R. Thurik. These authors contributed 13.11 % of the 68,283 citation counts. The number of articles and credited citation counts of authors are shown in Fig. 3. The correlation coefficient between the number of articles and the credited citation counts of authors was approximately 0.63, which indicates that a greater number of entrepreneurship articles published by an author resulted in a higher citation count (approximately 60 %). The top 19 authors with the most credited citation count (greater than 250) are shown in Table 6. The number of published articles for each author is also shown in parenthesis. Most of the authors have published more than 10 articles. Table 6 also presents the authors who have published less than 10 articles but have contributed a significant credited citation count. These authors include C. Zott, Benjamin M. Oviatt, Jay B. Barney, R. Amit, Mike W. Peng, and Kathleen M. Eisenhardt. The numbers explain certain peaks that are inconsistent with the trend of the number of published authors.

**Table 6:** Most Active Researchers’ Credit of cited times (1996–Jun. 2012)

	Researcher	Credit of cited times (No. of articles)	% of 68,283 cited times (5,476 articles)
1.	Shane, S	2,118 (36)	3.10
2.	Baron, RA	745 (26)	1.09
3.	Zahra, SA	728 (33)	1.07
4.	Venkataraman, S	622 (10)	0.91
5.	Shepherd, DA	534 (44)	0.78
6.	Busenitz, LW	436 (14)	0.64
7.	Wright, M	389 (56)	0.57
8.	McDougall, Patricia P.	365 (16)	0.53
9.	Audretsch, David	334 (39)	0.49
10.	Zott, C	287 (6)	0.42
11.	Oviatt, Benjamin M.	282 (9)	0.41
12.	Honig, B	282 (10)	0.41
13.	Davidsson, P	282 (13)	0.41
14.	Barney, Jay B.	271 (6)	0.40
15.	Amit, R	259 (4)	0.38
16.	Peng, Mike W.	258 (5)	0.38
17.	Eisenhardt, Kathleen M.	255 (6)	0.37
18.	Sorenson, O	255 (10)	0.37
19.	Wiklund, J	252 (17)	0.37
	7,386 authors	<252	86.89
	Total authors: 7,405		

### Most frequently cited entrepreneurship articles

The 5,476 entrepreneurship research articles were ranked based on each article’s total citation count in the ISI WOS database. The most cited entrepreneurship articles published between 1996 and June 2012 are shown in Table 7, which also shows the names of authors, citation count, article titles, and journal names. Concerning the “why, when, and how” in three sets of entrepreneurship questions: (1) opportunities for the creation of goods and services come into existence, (2) some people and not others discover and exploit these opportunities, and (3) different modes of action are used to exploit entrepreneurial opportunities (Shane and Venkataraman 2000). In the article “The promise of entrepreneurship as a field of research” (Shane and Venkataraman 2000), the authors argued that numerous people have had difficulty in identifying the field and the domain, thus undermining the distinct contribution of entrepreneurship. Therefore, the authors conducted a study on the different social science disciplines and applied fields of business to establish a conceptual framework for the entrepreneurship field employed in previous research. With the framework, a set of empirical phenomena is explained, and a set of outcomes is predicted. This article was cited 1,063 times and ranked first.

**Table 7:** The top-seven most frequently-cited entrepreneur articles (1996–Jun. 2012)

Rank	Authors	Times cited	Article title	Journal
1	Shane and Venkataraman (2000)	1,063	The promise of entrepreneurship as a field of research	Academy of management review
2	Shane (2000)	559	Prior knowledge and the discovery of entrepreneurial opportunities	Organization science
3	Eisenhardt and Schoonhoven (1996)	459	Resource-based view of strategic alliance formation: Strategic and social effects in entrepreneurial firms	Organization science
4	Amit and Zott (2001)	418	Value creation in e-business	Strategic management journal
5	Blanchflower and Oswald (1998)	367	What makes an entrepreneur?	Journal of labor economics
6	Busenitz and Barney (1997)	352	Differences between entrepreneurs and managers in large organizations: Biases and heuristics in strategic decision-making	Journal of business venturing
7	Davidsson and Honig (2003)	314	The role of social and human capital among nascent entrepreneurs	Journal of business venturing

The article “Prior knowledge and the discovery of entrepreneurial opportunities” (Shane 2000) was cited 559 times and ranked second. This article portrays the recognition of opportunities created from technological innovations. Using empirical data adopted from Austrian economics, this study portrays the recognition of such opportunities as distinctive cognitive achievements, the accomplishment of which is conditioned by the prior experience and education of an entrepreneur. In this study, the author also cited multiple opportunities that can arise from a single innovation in his in-depth case studies.

The article “Resource-based view of strategic alliance formation: Strategic and social effects in entrepreneurial firms” (Eisenhardt and Schoonhoven 1996) attempts to capture the strategic and social factors that propel numerous firms to form alliances. This study focuses on entrepreneurial semiconductor firms and develops findings by extending the resource-based view of firms in alliance formation and by examining the resulting hypotheses using product development alliances. This article combines alternative social and strategic explanations for alliance formation. Consistent with these explanations, the authors argued that alliances form when firms are in either of three vulnerable strategic positions: (1) they are competing in emerging industries, (2) they belong to highly competitive industries, or (3) they are attempting pioneering technical strategies. The authors also argued that alliances form when firms are in strong social positions, such that they are led by large, experienced, and well-connected top management teams. Therefore, the fundamental logic of alliance formation includes strategic needs and social opportunities. They also concluded that failure to include serial and strategic explanations results in an impoverished view of alliance formation. This article was cited 459 times and was ranked third.

The article “Value creation in e-business” (Amit and Zott 2001) was cited 418 times and was ranked fourth. By examining 59 American and European e-businesses, the authors explored the theoretical foundations of value creation in e-business. Grounded on data acquired from case study analyses and referring to the related theory in entrepreneurship and strategic management, they developed a model of the sources of value creation and suggested that the value creation potential of e-businesses is centered on the dimensions of efficiency, complementarities, lock-in, and novelty.

The article “What makes an entrepreneur?” (Blanchflower and Oswald 1998) was cited 367 times and was ranked fifth. Results imply that the probability of self-employment depends positively upon whether the individual ever received an inheritance or gift. Raising capital is the main problem for potential entrepreneurs in their directly questioned interview surveys. In their study, the authors also found that self-employed individuals report higher levels of job and life satisfaction than employees. They also argued that after testing, childhood psychology strongly correlated with later self-employment.

The article “Differences between entrepreneurs and managers in large organizations: Biases and heuristics in strategic decision-making” (Busenitz and Barney 1997) was cited 352 times and was ranked sixth. This article explores differences between entrepreneurs and managers in large organizations based on responses from 124 entrepreneurs and 95 managers. The differences were examined with respect to two biases and heuristics: overconfidence and representativeness. Results of logistic regression analysis indicate that in large organizations, entrepreneurs are more susceptible to use decision-making biases and heuristics than managers.

The seventh-ranked article “The role of social and human capital among nascent entrepreneurs” (Davidsson and Honig 2003) examines nascent entrepreneurship by comparing individuals engaged in nascent activities (380 nascent entrepreneurs) with a control group (608 non-entrepreneurs). Both groups were drawn from a sample of a general population of 30,427 of Swedish adults. Findings indicate that bridging and bonding social capital is a robust predictor for nascent entrepreneurs as well as for advancing through the start-up process. Results also support the view that human capital predicts entry into a nascent entrepreneurship but barely carries the start-up process toward a successful completion. This article was cited 314 times. The aforementioned seven articles were cited 3,532 times, comprising approximately 5.2 % of the 68,283 total citation counts in the 5,476-article pool.

### Chi square tests of Lotka’s Law for authors’ publications

The plot of the number of articles versus the number of authors is shown in solid line in Fig. 3. As expected, the diagram indicates a sharp decreasing curve. Considering for Lotka’s property, Table 8 presents a result of the Chi square test of Lotka’s Law for entrepreneurship literature. We considered three samples, namely, with 100/*b* ^2^ (%), with 100/*b* ^2.6^ (%), and the grouped case (in percentage). Based on the Chi square values, Lotka’s original suggestion of *x*/*b* ^2^ does not apply. However, the *x*/*b* ^2.6^ prediction is quite close.

**Table 8:** Chi squared test of Lotka’s Law

Contribute *n* papers	Predicted 100/*n* ^2^ (%)	Predicted 100/*n* ^2.6^ (%)	No. of authors	% of *n* = 1
1	100	100	5,773	100
2	25	15.389	892	15.451
3	11.11	5.150	315	5.456
4	6.25	2.368	164	2.841
5	4.00	1.297	56	0.970
6	2.78	0.792	59	1.022
7	2.04	0.523	30	0.520
8	1.56	0.364	38	0.658
9	1.23	0.265	15	0.260
10	1.00	0.200	13	0.225
11	0.83	0.154	9	0.156
12	0.69	0.122	5	0.087
13	0.59	0.098	7	0.121
14	0.51	0.080	3	0.052
15	0.44	0.067	6	0.104
16	0.39	0.056	3	0.052
17	0.35	0.048	4	0.069
18	0.31	0.041	2	0.035
19	0.28	0.035	1	0.017
20	0.25	0.031	1	0.017
22	0.21	0.024	1	0.017
24	0.17	0.019	2	0.035
26	0.15	0.015	1	0.017
33	0.09	0.008	1	0.017
36	0.08	0.006	1	0.017
39	0.07	0.005	1	0.017
44	0.05	0.004	1	0.017
56	0.03	0.002	1	0.017

Chi squared value for 100/*n* ^2^: 18.73, Chi squared value for 100/*n* ^2.6^: 0.68

## Discussion and limitation

This study represents an effort to measure the contributions of individual and institutional contributors to entrepreneurship research from 1996 to June 2012. Approximately 5,476 articles of acceptable scope, credibility, and significance were identified. The influence of individual articles and individual authors on the entrepreneurship literature was also assessed based on the SCI/SSCI citation analysis. The implications of this study are discussed with the hope of offering several concluding remarks about entrepreneurship studies.

First, entrepreneurship research has attracted a significant number of researchers during the study period covering 16.5 years. Based on our findings, researchers from North America (the USA and Canada), Europe (England, Germany, the Netherlands, Spain, Sweden, Italy, France, Scotland, Belgium, and Switzerland), and Australia have made the most contributions to this field. The authors conclude that scholars are more likely to conduct research on entrepreneurship when more developed countries are present in a particular area.

Second, a number of major journals published the most number of entrepreneurship research articles. These journals include the Journal of Business Venturing and Small Business Economics, each of which has published more than 300 articles related to entrepreneurship, and both continue to contribute articles today. The two also contributed more than 10 % of the total number of articles among the 522 journals. However, limited to the algorithm and the keywords adopted, some mainstream journals do not show up in the study. We do not intend to ignore the contributions of these journals or to disregard their contributions. These journals include Journal of Technology Transfer, which published 38 articles; Journal of Management published, which published 35 articles; Family Business Review, which published 33 articles; Academy of Management Journal, which published 31 articles; and Administrative Science Quarterly, which published 10 articles in our survey period. These journals still contribute significantly in this field.

Third, several main contributors have contributed to the field from 1996 to June 2012, and their scholarship has had a significant influence on those who classify themselves as “entrepreneur” researchers. The participation of more new scholars in this field would be very helpful. These findings will hopefully enable authors to conduct future research on the further development of entrepreneurship.

Fourth, this study demonstrates that the number of articles related to entrepreneurship from 1996 to June 2012 is growing. An up-trend slope is noted, which indicates that the influence of entrepreneurship is still on the rise. We expect that these findings can help and encourage authors to develop more studies about entrepreneurship in the future.

Several limitations are noted in this study in the attempt to provide reasons for our findings. First, this work does not consider non-SCI/SSCI journals. Notably, we did not intend to ignore the contributions of other journals or to disregard the contributions of researchers published in those journals. Second, articles not cited in the ISI WOS database and published before 1996 were not considered in this study. Focusing on the given period was essential in finding relevant and updated entrepreneur researchers. Furthermore, the SSCI citation count does not explain citations by articles published in non-SSCI-indexed journals, although such are conventional and well accepted in the academic field. Consequently, the reported citation counts in this study might underestimate the total number of citations of an article in the academic literature. Finally, the method by which we ranked the most cited articles or calculated the credit of cited times for authors may be inappropriate. For example, if there are two authors, each gets credit for only 1/2 of the citations. As a result, schools and individuals with more collaborative work are less likely to be highly ranked than those with more individual work. Consequently, authors with more overall citations may be “off” the list. For example, authors such as Aldrich continually published several main studies and contributed substantially to the development of entrepreneurship. However, given the limitation of the calculation of credit of cited times adopted, his eight articles (ranked 79th) and 105 cited times (ranked 94th) was not listed in the tables. This is a factors that does not appear to be in the co-authors’ best interest to enter.
